# PROTEIN PHOSPHATASE 2C08, a Negative Regulator of Abscisic Acid Signaling, Promotes Internode Elongation in Rice

**DOI:** 10.3390/ijms241310821

**Published:** 2023-06-28

**Authors:** Jaeeun Song, Eunji Ga, Sangkyu Park, Hyo Lee, In Sun Yoon, Saet Buyl Lee, Jong-Yeol Lee, Beom-Gi Kim

**Affiliations:** 1 Metabolic Engineering Division, National Institute of Agricultural Sciences, Rural Development Administration, Jeonju 54874, Republic of Korea; icanje@korea.kr (J.S.); pak2779@korea.kr (S.P.);; 2 Gene Engineering Division, National Institute of Agricultural Sciences, Rural Development Administration, Jeonju 54874, Republic of Korea

**Keywords:** ABA signaling, PP2CA, GA signaling, GA biosynthesis

## Abstract

Clade A protein phosphatase 2Cs (PP2CAs) negatively regulate abscisic acid (ABA) signaling. Here, we investigated the functions of OsPP2CAs and their crosstalk with ABA and gibberellic acid (GA) signaling pathways in rice (*Oryza sativa*). Among the nine OsPP2CAs, OsPP2C08 had the highest amino acid sequence similarity with OsPP2C51, which positively regulates GA signaling in rice seed germination. However, *OsPP2C08* was expressed in different tissues (internodes, sheaths, and flowers) compared to *OsPP2C51*, which was specifically expressed in seeds, and showed much stronger induction under abiotic stress than *OsPP2C51*. Transgenic rice lines overexpressing *OsPP2C08* (OsPP2C08-OX) had a typical ABA-insensitive phenotype in a post-germination assay, indicating that OsPP2C08, as with other OsPP2CAs, negatively regulates ABA signaling. Furthermore, OsPP2C08-OX lines had longer stems than wild-type (WT) plants due to longer internodes, especially between the second and third nodes. Internode cells were also longer in OsPP2C08-OX lines than in the WT. As GA positively regulates plant growth, these results suggest that OsPP2C08 might positively regulate GA biosynthesis. Indeed, the expression levels of GA biosynthetic genes including gibberellin 20-oxidase (OsGA20ox4) and Ent-kaurenoic acid oxidase (OsKAO) were increased in OsPP2C08-OX lines, and we observed that GIBBERELLIN 2-OXIDASE 4 (*OsGA2ox4*), encoding an oxidase that catalyzes the 2-beta-hydroxylation of several biologically active GAs, was repressed in the OsPP2C08-OX lines based on a transcriptome deep sequencing and RT-qPCR analysis. Furthermore, we compared the accumulation of SLENDER RICE 1 (SLR1), a DELLA protein involved in GA signaling, in OsPP2C08-OX and WT plants, and observed lower levels of SLR1 in the OsPP2C08-OX lines than in the WT. Taken together, our results reveal that OsPP2C08 negatively regulates ABA signaling and positively regulates GA signaling in rice. Our study provides valuable insight into the molecular mechanisms underlying the crosstalk between GA and ABA signaling in rice.

## 1. Introduction

Phytohormones are chemical messengers that regulate cellular responses at very low concentrations. Plant development is regulated by seven major phytohormones: auxin, gibberellic acid (GA), cytokinin, ethylene, abscisic acid (ABA), brassinosteroids, and strigolactone. ABA negatively modulates plant growth and germination, and increases plant tolerance to osmotic stress, cold, and drought [1,2,3]. ABA biosynthesis begins in plastids and ends in the cytosol. As an immediate stress messenger, glucose-bound inactive ABA accumulates in the endoplasmic reticulum before being converted to its active form. A rapid increase of ABA concentrations in plant cells serves as a signal under various abiotic stress conditions [4,5]. Intracellular recognition of ABA is initiated by the ABA receptors PYRABACTIN RESISTANCE 1/PYR1-LIKE/REGULATORY COMPONENTS OF ABA RECEPTOR (PYR/PYL/RCAR) and Clade A protein phosphatase 2Cs (PP2CAs). PP2CA negatively regulates ABA signaling [6,7]. Indeed, in the presence of ABA, an ABA receptor–ABA–PP2CA trimeric complex assembles and SNF1-related protein kinase 2s (SnRK2s), whose kinase activity is inhibited by PP2CA, are activated. Activated SnRK2s phosphorylate downstream basic leucine zipper (bZIP) transcription factors and anion channels, such as SLOW ANION CHANNEL-ASSOCIATED 1 (SLAC1), to initiate ABA signaling.

Bioactive GAs positively regulate plant growth and development. GA biosynthesis begins in plastids and ends in the cytosol [8]. When the intracellular GA concentration rises, DELLA proteins, which negatively regulate GA signaling, act immediately downstream of the GA receptor GIBBERELLIN INSENSITVE DWARF 1 (GID1) [3,9]. As in other proteosome degradation pathways, the first step in DELLA degradation is a specific interaction between GID1-GA-DELLA protein complexes and F-box proteins, a component of the SCF (Skp1, Cullin, F-box proteins) E3 ubiquitin ligase complex [10,11,12].

ABA and GA function antagonistically in plants during various developmental decisions, such as seed dormancy and germination, root growth, leaf development, and flowering time, and in response to environmental cues such as light, temperature, and stress [13,14,15,16,17]. For example, the NUCLEAR FACTOR-Y C (NF-YC), RGA-LIKE 2 (RGL2), and ABA INSENSITIVE 5 (ABI5) modules integrate GA and ABA signaling pathways during seed germination. As another example, the Chinese cabbage (*Brassica rapa* subsp. *pekinensis*) NAC transcription factor BrNAC041 mediates ABA-antagonized GA accumulation during ABA-induced leaf senescence [18,19].

HONSU, a seed-specific PP2CA, regulates seed dormancy by inhibiting ABA signaling, and activates GA signaling to promote seed germination in Arabidopsis (*Arabidopsis thaliana*) [20]. SbABI4 and SbABI5 from sorghum (*Sorghum bicolor*) bind to the *GIBBERELLIN 2-OXIDASE 3* (*SbGA2ox3*) promoter in vitro [21]. In rice (*Oryza sativa*), *OsPP2C51* represses the bZIP transcription factor OsbZIP10 (a homolog of Arabidopsis ABI5) via dephosphorylation to promote seed germination, and overexpression of *OsPP2C51* leads to increased expression of α-amylase genes and increased activity of the encoded enzyme [22]. Thus, OsPP2C51 is a positive regulator of GA signaling that has been implicated in crosstalk between ABA and GA signaling.

Here, we investigated the functions of OsPP2C08 and its roles in crosstalk between GA and ABA signaling pathways. We overexpressed *OsPP2C08* (OsPP2C08-OX) in rice. OsPP2C08-OX lines grew taller than the wild type (WT), suggesting that OsPP2C08 promotes stem growth by positively regulating GA signaling. Furthermore, OsPP2C08-OX lines had a typical ABA-insensitive phenotype, indicating that OsPP2C08 negatively regulates ABA signaling. These results shed light on the crosstalk between ABA and GA signaling in rice.

## 2. Results

### 2.1. Expression Pattern of OsPP2C08 and Subcellular Localization of OsPP2C08

**Figure 1:**
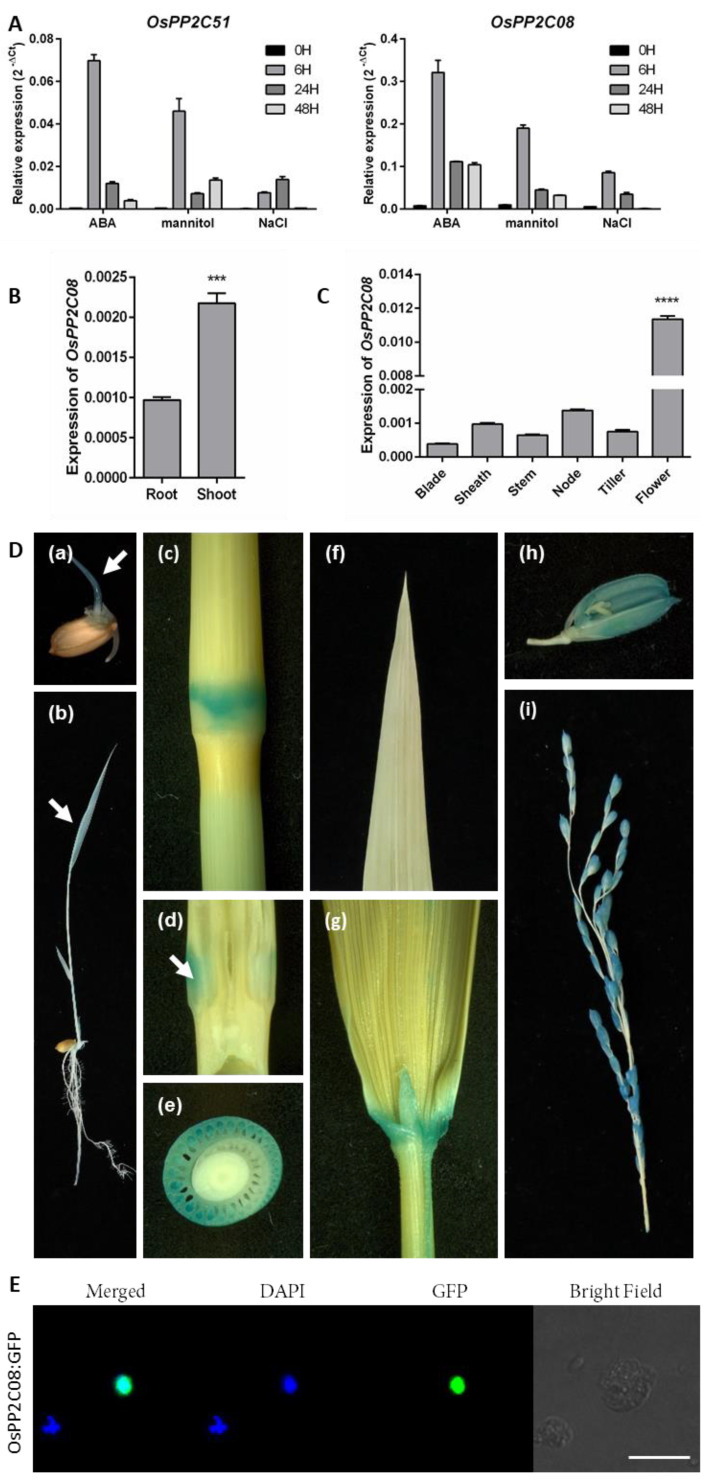
*OsPP2C08* expression profile in different rice tissues and OsPP2C08 subcellular localization. (**A**) *OsPP2C51* and *OsPP2C08* expression in response to abiotic stresses. All values were determined by RT−qPCR and normalized to *UBIQUTIN* 5 levels using the ∆Ct method. (**B**) *OsPP2C08* expression levels in shoots and roots of young rice seedlings by RT−qPCR and normalized to *UBIQUTIN* 5 levels, using the ∆Ct method (Student’s *t*-test and nonparametric test with unpaired test, ***: *p* < 0.001). (**C**) *OsPP2C08* expression levels in mature rice tissues (leaf blade, leaf sheath, stem, tiller, and flower) by RT−qPCR and normalized to *UBIQUTIN* 5 levels, using the ∆Ct method (one-way ANOVA with Tukey’s test, ****: *p* < 0.0001). Data are means ± SEM (*n* = 3). (**D**) Histochemical GUS staining analysis of *proOsPP2C08:GUS* transgenic rice seedlings and plants. Two-day-old seedlings (**a**), two-week-old seedlings (**b**), nodes (**c**), longitudinal section of a node (white arrow indicates the intercalary meristem) (**d**), horizontal section of a node (**e**), leaf blade (**f**), lamina joint (**g**), flower (**h**), and panicle (**i**). (**E**) Subcellular localization of *OsPP2C08*−*GFP* in a rice protoplast. OsPP2C−GFP was used at 10 μg per transfection. Exposure time of GFP fluorescence was 200 ms. Scale bars, 10 μm.

Among the nine rice PP2CAs, OsPP2C08 showed the highest amino acid sequence similarity to OsPP2C51, and the two proteins clustered in the same subgroup in our phylogenetic analysis (Figure S1). These two proteins were induced by abiotic stress, as are the other *OsPP2CA* genes, but *OsPP2C08* was expressed at much higher levels than *OsPP2C51* [22] (Figure 1A). *OsPP2C08* and *OsPP2C51* were rapidly induced by ABA, mannitol, and NaCl treatments; their expression levels peaked at 6 h after the onset of stress, then gradually decreased (Figure 1A). We detected *OsPP2C08* expression in various tissues by reverse-transcription quantitative PCR (RT-qPCR) analysis. At the young seedling stage, OsPP2C08 was expressed two-fold higher in the shoot than in roots (Figure 1B). In mature rice, *OsPP2C08* was expressed at higher levels in nodes and flowers than in other tissues (Figure 1C).

To further explore the *OsPP2C08* expression pattern, we generated transgenic rice plants expressing the *GUS* reporter gene driven by the *OsPP2C08* promoter (*proOsPP2C08:GUS*). Histochemical staining for GUS activity showed that GUS accumulates to higher levels in hypocotyl tissues than in roots at 2 days after germination (Figure 1D(b)), which was consistent with our RT-qPCR result. At the flowering stage, we observed high GUS activity in the lemma, palea, lamina joints, and internodes (Figure 1D). In particular, we detected strong GUS activity in the intercalary meristem in the internode (Figure 1D(c,d)).

We also explored the subcellular localization of OsPP2C08. We introduced a fusion construct consisting of *OsPP2C08-GFP* (*GREEN FLUORESCENCE PROTEIN*) driven by the cauliflower mosaic virus (CaMV) 35S promoter into rice protoplasts. The OsPP2C08-GFP fusion protein colocalized with the nuclear staining agent DAPI, indicating that OsPP2C08 is a nucleus-localized protein (Figure 1E).

### 2.2. Overexpression of OsPP2C08 in Rice Causes ABA Insensitive Phenotypes

To study the function of OsPP2C08, we generated 15 transgenic OsPP2C08-overexpressing (OsPP2C08-OX) rice lines and selected 3 lines (#1, #2, and #4) based on high *OsPP2C08* expression level for further analysis.

**Figure 2:**
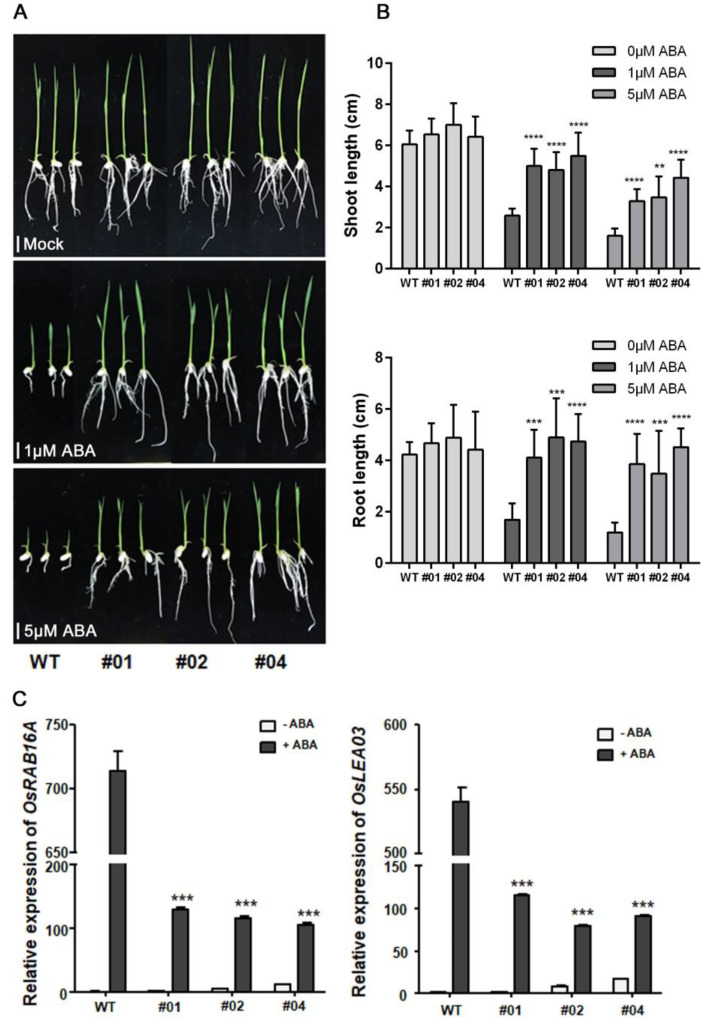
ABA−insensitive phenotypes of transgenic rice overexpressing *OsPP2C08*. (**A**) OsPP2C08-OX(#01, #02, #04) and WT (Wild type) seedlings were transferred 3 days after germination to half-strength MS medium without ABA (**top panel**), with 1 μM ABA (**middle panel**) or with 5 μM ABA (**bottom panel**). Photographs show representative seedlings at 4 days (**top panel**) or 5 days (**middle and bottom panel**) after transfer. Scale bar, 1 cm. (**B**) Shoot growth rate (**upper panel**) and root growth rate (**bottom panel**) of OsPP2C08−OX and WT plants grown with or without ABA (*n* = 12). Data are means ± SEM. (one-way ANOVA with Tukey’s test, **: *p* < 0.01, ***: *p* < 0.001, ****: *p* < 0.0001). (**C**) Relative *OsRAB16A* and *OsLEA03* expression in OsPP2C08-OX seedlings at 6 h after 5 μM ABA treatment. Gene expression levels were determined by RT−qPCR, and normalized to *UBIQUTIN* 5 levels using the ∆∆Ct method. Data are means ± SEM (one-way ANOVA with Tukey’s test, ***: *p* < 0.001).

First, we investigated the growth of the three OsPP2C08-OX lines in response to ABA treatment in a post-germination assay. When grown on half-strength MS medium without ABA, the transgenic seedlings were at maximum 16% taller than the WT, but the height difference between OsPP2C08-OX and WT seedlings became more pronounced under ABA treatment. Under 5 μM ABA, stem and root growth were strongly suppressed in the WT, but were much less affected in the OsPP2C08-OX lines (Figure 2A).

We investigated the expression levels of the ABA-responsive genes *RESPONSIVE TO ABSCISIC ACID 16A* (*OsRAB16A*) and *LATE EMBRYOGENESIS ABUNDANT 3* (*OsLEA3*) to monitor the inhibition of ABA signaling in the OsPP2C08-OX lines. Under the 5 μM ABA treatment, *OsRAB16A* and *OsLEA3* were dramatically induced in both genotypes, but their expression levels were about 6-fold lower in the transgenic lines compared to WT (Figure 2C), indicating that OsPP2C08 negatively regulates ABA signaling.

### 2.3. OsPP2C08 Promotes Internode Elongation in Rice

**Figure 3:**
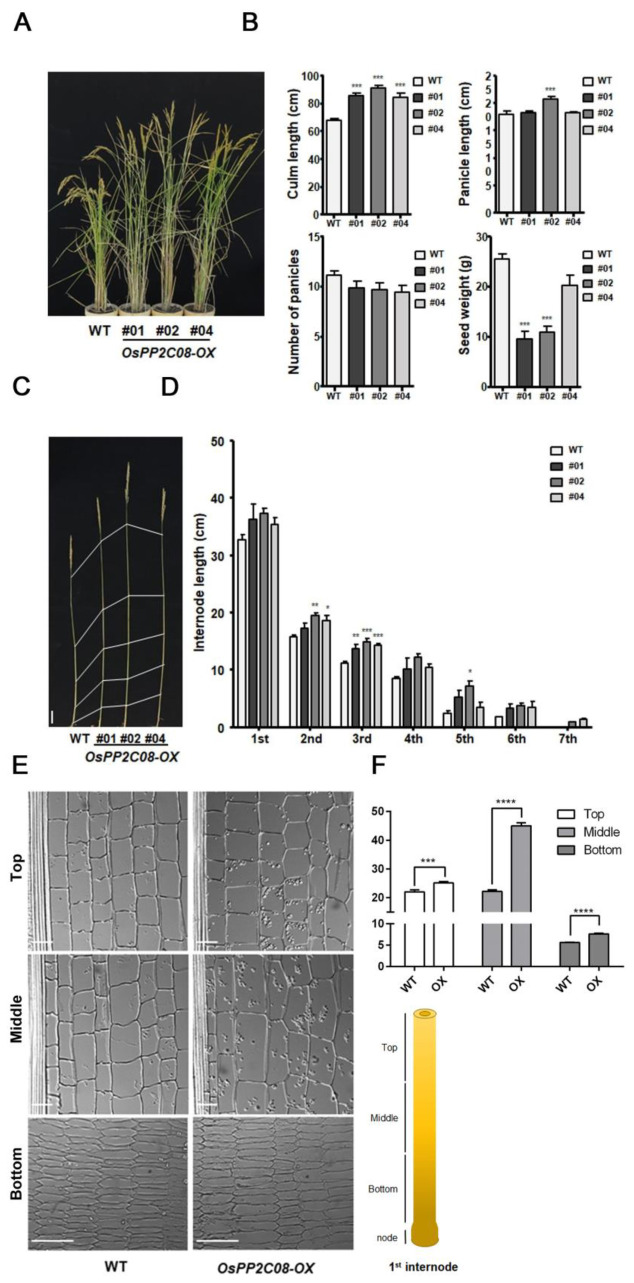
Internode elongation phenotypes in the OsPP2C08−OX lines. (**A**) Representative photograph of mature OsPP2C08-OX and WT plants grown in a paddy field. (**B**) Culm length, panicle length, number of panicles, and total seed weight of OsPP2C08-OX and WT plants (*n* = 7). Data are means ± SEM. (one-way ANOVA with Tukey’s test, ***: *p* < 0.001). (**C**) Representative photograph of rice stems in OsPP2C08-OX and WT plants grown in a paddy field. Scale bar, 10 cm. (**D**) Internode length in OsPP2C08-OX and WT plants (*n* = 7). Data are means ± SEM. Asterisks indicate a significant difference (one-way ANOVA with Tukey’s test, *: *p* < 0.05, **: *p* < 0.01 ***: *p* < 0.001). (**E**) Representative micrographs of cell walls in the first internode of OsPP2C08-OX and WT plants grown in a paddy field. Scale bar, 20 μm. (**F**) Cell length in OsPP2C08-OX and WT plants (*n* = 5). Data are means ± SEM (*t*-test and parametric test with paired test, ***: *p* < 0.001, ****: *p* < 0.0001).

We grew OsPP2C08-OX plants in the paddy field, and compared their agricultural traits to those of rice cultivar Dongjin (WT) plants. The transgenic lines were 20% taller than the WT (Figure 3A). The OsPP2C08-OX lines had fewer panicles, and panicle length was similar to that of the WT, except for OX line #2 (Figure 3B). These results were consistent throughout the three years of our study. Furthermore, panicle number and grain weight were lower in the transgenic lines under normal paddy field conditions, and flowering occurred a few days earlier than in the WT (Table S2).

To explore the cause of the increased plant height in OsPP2C08-OX lines, we measured internode number and length in mature plants. The internodes were longer in all OsPP2C08-OX lines compared to WT plants (Figure 3C,D).

To determine whether the increased plant height was caused by a variation in the number of cells or cell length, we divided the fully elongated first internode of rice plants grown in paddy fields into three regions, and sectioned each longitudinally [23]. Then, we measured cell lengths and counted the number of cells (Figure 3E,F). The middle segment of internode cells of OsPP2C08-OX plants were significantly longer than those of the WT (Figure 3F). These results indicate that the increased internode length in the transgenic plants was caused by changes in cell elongation, and we speculated that this phenotype may be related to enhanced GA signaling.

### 2.4. OsPP2C08 Overexpression Positively Regulates the Expression of GA Biosynthesis Genes

**Figure 4:**
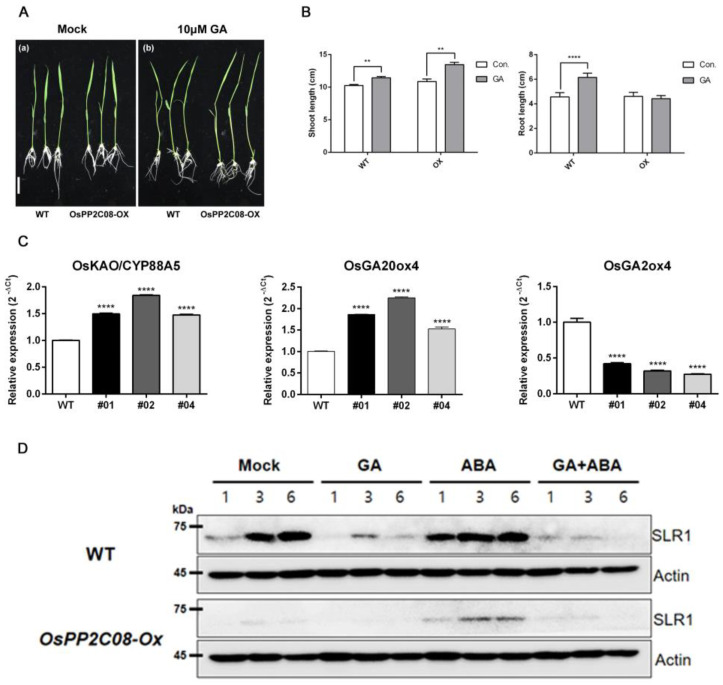
GA−sensitive phenotype of the OsPP2C08-OX lines(#01, #02, #04), expression analysis of GA biosynthesis genes, and immunoblotting for SLR1. (**A**) OsPP2C08-OX and WT seedlings were transferred at 3 days after germination to half-strength MS medium containing 10 μM GA. Control (**a**), 10 μM GA (**b**). Representative photograph of seedlings 4 days after transfer. Scale bar, 2 cm. (**B**) Shoot growth rate (**left panel**) and root growth rate (**right panel**) of OsPP2C08-OX and WT seedlings grown with or without GA (*n* = 10). Data are means ± SEM. (one-way ANOVA with Tukey’s test, **: *p* < 0.01 ****: *p* < 0.0001). (**C**) Relative expression of GA biosynthesis genes in 7-day-old OsPP2C08-OX(#) and WT seedlings, as determined by RT−qPCR. Data are means ± SEM of 3 biological replicates. (one-way ANOVA with Tukey’s test, ****: *p* < 0.0001). (**D**) Immunoblotting for SLR1.

Phenotypes displayed by the OsPP2C08-OX lines might be positive to GA signaling. Thus, to explore GA signaling in the OsPP2C08-OX lines, we performed a post-germination assay using a 10-μM GA treatment. Under this GA treatment, the shoots of WT and OsPP2C08-OX seedlings were longer, but root elongation was inhibited in the transgenic seedlings compared to the WT (Figure 4A,B).

Next, we conducted transcriptome deep sequencing (RNA-seq) to dissect the expression profile of GA biosynthetic genes in the transgenic lines. We determined that *GA20ox4* and *ENT-KAURENOIC ACID OXIDASE* (*KAO*), key regulators of GA biosynthesis, are significantly upregulated, while *GA2ox4*, encoding an enzyme that catalyzes active GA, was downregulated in the transgenic lines compared to the WT (Table 1). We validated these results by RT-qPCR using *OsUBI05* as an internal control (Figure 4C).

SLR1, previously named OsGAI, is the only DELLA protein in rice. GA-induced degradation of SLR1 is a key step in the GA signaling pathway in this species. To further explore the GA pathway in the transgenic lines, we investigated the time-dependent degradation of SLR1 in WT and OsPP2C08-OX seedlings grown on half-strength MS medium for 8 days, and then transferred to half-strength MS containing 50 μM GA_3_ or 50 μM ABA. We detected less SLR1 protein in the OsPP2C08-OX seedlings than in the WT under the mock, ABA-, and GA_3_-treated conditions. Furthermore, SLR1 was degraded in the GA3-treated WT seedlings, and SLR was almost undetectable in the GA_3_-treated OsPP2C08-OX seedlings. As previously described by Kim S. et al., SLR1 was more stable in the presence of ABA [24]. When seedlings were treated with ABA and GA_3_, the SLR1 signal in the OsPP2C08-OX lines was weaker than when treated with ABA alone (Figure 4D). Taken together, our results suggest that *OsPP2C08* overexpression activates GA signaling by activating GA biosynthesis.

## 3. Discussion

In this study, we report that *OsPP2C08*, a PP2CA, functions as a positive regulator of GA signaling in terms of plant growth, as well as a typical negative regulator of ABA signaling. GA biosynthesis genes were induced and DELLA protein stability was reduced in OsPP2C08-OX plants, even though we did not clarify the molecular mechanisms by which OsPP2C08 activates GA signaling by activating GA biosynthesis.

The amino acid sequence of OsPP2C08 is similar to that of OsPP2C51, a positive regulator of GA signaling [22], and plants overexpressing their encoding genes have similar phenotypes. However, *OsPP2C51* was transcribed specifically in seeds and might function only in seed germination, whereas *OsPP2C08* was also expressed in other tissues, such as nodes, flowers, and leaves. Thus, the biochemical characteristics of *OsPP2C08* might be similar to those of *OsPP2C51*. However, their differences in gene expression patterns might underlie their different cellular functions and resulting phenotypes.

PP2CAs are key regulators that interact with SnRK2s to negatively regulate ABA signaling and the plant’s adaptive response to various stresses [25,26]. Several PP2CAs also function as positive regulators of GA signaling. Overexpression of *OsPP2C51* induced α-amylase gene expression and accelerated seed germination [22]. When *PtrHAB2*, a PP2CA of *Populus trichocarpa* was overexpressed, the resulting transgenic plants grew faster and taller than the control plants [27]. Overexpression of the PP2CA *OsPP2C09* promotes plant growth and results in increased grain yield, whereas gene editing of *OsPP2C09* suppressed growth and reduced the grain yield [28]. Thus, PP2CA might be a key regulator of crosstalk between ABA and GA signaling.

The molecular mechanisms by which PP2CAs are involved in ABA signaling are well established. PP2CAs function as co-receptors with the ABA receptors PYR/RCARs to form a PP2C-ABA-PYR/RCAR complex that acts as a switch in ABA signaling. However, the molecular mechanism by which OsPP2CAs transduce GA signaling is unclear. The rice genome encodes around 1429 kinases and 132 phosphatases [29,30]. As there are fewer phosphatases than kinases, phosphatases may regulate several kinases or signaling components to play diverse roles in plants. Thus, PP2CAs might interact with diverse partners in signaling pathways. Indeed, it was reported that PP2CAs interact with several other signaling components in addition to SnRKs in ABA signaling. *OsPP2C51* dephosphorylates and directly interacts with OsbZIP10 and *OsPP2C09*, which interacts with DRE-binding (DREB) transcription factors to activate them, as well as with the RING E3 ligase OsRF1 [22,31]. CaPP2CAs interact with CaMEKKs, which are phosphorylated by CaSnRK2.6. ABI1 interacts with the 1-aminocyclopropane-1-carboylate synthases (ACSs), which catalyze ethylene biosynthesis and modulate the protein stability [32,33,34]. ABI1 interacts with CASEIN KINASE 1-LIKE PROTEIN2 (CKL2) to regulate actin reorganization in stomatal closing. ABA Hypersensitive germination 1 (AHG1), a PP2CA, interacts with DELAY OF GERMINATION 1 (DOG1).

Recent studies on the molecular mechanisms of seed dormancy controlled antagonistically by GA and ABA have established that DOG1 is a critical regulator that determines the intensity of primary seed dormancy, and that DOG1 interacts with the PP2CAs AHG1 and AHG3 and impairs their phosphatase activity, activating ABA signaling and extending seed dormancy [35,36].

OsABA2 mutant, which is involved in ABA biosynthesis pathways, was reported to have lower ABA contents and higher GA contents than the control plant. The mutant showed enhanced growth of the stem and leaf [37]. There were several reports for growth enhancement in plants overexpressing PP2CAs. Miao et al. (2020) [28] reported that OsPP2C09-Ox plants were taller, with greater fresh and dry shoot weight, and Min et al. (2021) [7] also described that the seedling growth was enhanced in OsPP2C09-Ox plants. Endo et al. (2018) [6] reported that OsABIL ox lines enhanced seedling growth in low temperatures. ABA suppresses GA synthesis, the plant growth and gives tolerance to abiotic stresses. Thus, generally, the genes which interact directly with ABA signaling components and effect seriously ABA signaling might not only modulate ABA signaling, but also plant growth.

Several AP2 domain transcription factors, such as ABI4, OsAP2-39, and DDF1, can induce the gene expression of GA2ox7, which is the major enzyme to degrade GA. Additionally, the RAV1 transcription factor binds to promoter ABI4 and repress the gene expression of ABI4. SnRK2.3 phosphorylates the RAV1 and suppress the activity of RAV1. OsPP2C08, a clade A PP2C, can interact with SnRK2 family and overexpression of OsPP2C08 can activate the RAV1 and increase the GA contents in plant overexpressing OsPP2C08 [38,39].

Furthermore, the molecular mechanism by which GA and ABA antagonistically regulate plant growth was recently reported.

The Tiller Enhancer (TE) of APC/C^TE^ E3 ubiquitin ligase complex interacts with ABA receptor PYL/RCARs under high GA concentrations and degrades them [39,40,41]. Conversely, activated SnRK2s phosphorylate TE, inhibited APC/C^TE^, and stabilized the ABA receptors PYL/RCARs. Under high GA concentrations, APC/C^TE^ promotes the degradation of SHORT-ROOT1 (OsSHR1), a key factor promoting root growth, and MONOCULM1 (MOC1), a key factor promoting tiller generation. Conversely, under high ABA concentrations, OsSHR1 and MOC1 are activated and root growth and tiller generation is promoted.

These mechanisms can explain why our OsPP2C08-OX lines had fewer tillers, longer stems, and alterations in gene expression related to GA biosynthesis. Even though PP2CAs directly regulate the APC/C^TE^ pathway, PP2CAs regulate the activity of SnRK2, which is a central player in the pathway underlying the development of the GA phenotype in the OX plants.

Taken together, our study revealed that *OsPP2C08* overexpression in rice increases cell size and promotes stem growth, which are typical GA phenotypes in terms of growth regulation. Since PP2CAs negatively regulates ABA signaling, we propose that overexpressing PP2CAs suppresses ABA signaling and enhances GA signaling, thereby promoting growth in rice.

## 4. Materials and Methods

### 4.1. Generation of Transgenic Rice

To generate OsPP2C08-overexpressing transgenic rice (*Oryza sativa*), the *OsPP2C08* (Os01g46760) cDNA was amplified with Accu Prime *Pfx* DNA Polymerase. The resulting PCR product was cloned into the pENTR/D-Topo vector (Invitrogen, Carlsbad, CA, USA), and then transferred to the pGA2897 vector through Gateway LR recombination. The resulting *pUbi:OsPP2C08* construct, in which *OsPP2C08* was placed under the control of the constitutive maize (*Zea mays*) *Ubiquitin* promoter, was transformed into Agrobacterium (*Agrobacterium tumefaciens*) strain LBA4404 via electroporation. Transgenic rice plants were generated using the Agrobacterium-mediated co-cultivation method. Transformants were selected based on hygromycin resistance and transferred to the greenhouse [42].

For β-glucuronidase (GUS) reporter assays, the *OsPP2C08* promoter was cloned into the plant *GUS* expression vector pBGWSF7 and transformed into rice as described previously [31,42,43] Transgenic rice plants were selected based on phosphinothricin (PPT) resistance.

### 4.2. Total RNA Isolation and Reverse Transcription Quantitative Polymerase Chain Reaction (RT-qPCR)

Rice tissue samples (50–100 mg) were ground using a TisseLyser II (Quiane Inc., Germantown, WI, USA), and total RNA was isolated using an RNeasy Plant Mini Kit (Qiagen Inc., Hilden, Germany) according to the manufacturer’s manual. The purity and concentration of total RNA was measured using a Nanodrop spectrophotometer (Nanodrop ND-1000, Waltham, MA, USA). Total RNA was dissolved in DEPC-treated water, incubated at 65 °C for 10 min, and then transferred to ice. Then, 5 μg of total RNA was used to synthesize first-strand cDNA using an RNA to cDNA EcoDry Premix (Double Primed) (TAKARA, Kusatsu, Gunma, Japan). cDNA synthesis was performed at 42 °C for 60 min, and the enzyme was inactivated at 72 °C for 10 min. After cDNA synthesis, quantitative PCR was performed using TOPreal qPCR 2xPreMIX (SYBR Green with high ROX) (Enzynomics, Daejeon, Republic of Korea) on a StepOnePlus thermocycler (Applied Biosystems, Waltham, MA, USA). Rice *UBIQUITIN 5* (*OsUBI05*) was used as an internal control and relative gene expression was analyzed by the 2^−∆Ct^ or 2^−∆∆Ct^ method.

### 4.3. Protoplast Isolation and Subcellular Localization

For subcellular localization, the full-length coding sequence of *OsPP2C08* without the termination codon was amplified by PCR to generate the *35S:OsPP2C08-GFP* vector. *35S:OsPP2C08-GFP* was transfected into rice protoplasts by polyethylene glycol (PEG)-mediated transformation, as previously described [22,44]. After incubating the transfected protoplasts for 24 h at 28 °C, protoplasts were fixed by exchanging with W6 solution (154 mM NaCl, 125 mM CaCl_2_, 5 mM KCl, and 10 mM PIPES adjusted to pH 6.8) and 4% (*w*/*v*) paraformaldehyde. After a 2 h incubation, the buffer was exchanged for 4′,6′-diamidino-2-phenylindol (DAPI) staining buffer (W6 and 5 μg/mL DAPI). After a 10 min incubation and several washes with W6 solution, DAPI and green fluorescent protein (GFP) signals were captured using a Leica TCS SP8 laser scanning confocal microscope. The combinations of excitation/emission wavelength detection range for confocal microscopy were 488 nm (solid state laser)/493–530 bandpass for GFP and 405 nm (solid state laser)/410–493 bandpass for DAPI.

### 4.4. Post-Germination Assay

Surface-sterilized seeds were grown on half-strength Murashige and Skoog (MS) medium containing hygromycin B (40 mg/L, Duchefa, The Netherlands) for 3 days and transferred to half-strength MS containing ABA (2-cis-4-trans-abscisic acid, 98% synthetic, Sigma Aldrich, Burlington, MA, USA) or GA (90% gibberellin A3 basis, Sigma Aldrich, USA), then grown vertically from 4 to 6 days at 28 °C under 16 h light and 8 h darkness. After incubation at 28 °C, shoot and root lengths were measured.

### 4.5. Growth Conditions for Analyzing Agricultural Traits

Seeds of transgenic rice plants were germinated and grown for 1 week on water containing hygromycin B. After 2 days of acclimation in the greenhouse, young seedlings were transferred to pots (16 × 6 × 5.5 cm) filled with soil, and grown at 24–30 °C for 4 weeks in the greenhouse. Plants were transplanted into a paddy field in the middle of May, and seeds were harvested at the end of October annually for 3 years. To evaluate the agricultural traits of transgenic rice plants, total grain weight, culm and panicle length, number of panicles, and internode length were measured in 7 individual plants from each of 3 independent lines at maturity.

### 4.6. Histochemical GUS Assay and Measurement of Cell Size

For histochemical assays, surface-sterilized seeds were germinated and grown at 28 °C for approximately 2 weeks. Some seedlings were selected, and the remaining seedlings were transferred to soil and grown in a paddy field for 4 months, to yield mature tissue samples. Rice tissues were fixed in acetone for 10 s, and then GUS staining was performed using X-Glu staining solution (1 mM EDTA, 5 mM potassium ferricyanide, 100 mM sodium phosphate, 1% [*v*/*v*] Triton-X 100, and 1 mg/mL X-Glu) at 37 °C for 24 h. GUS-stained tissues were destained by incubation in 90% (*v*/*v*) ethanol until the chlorophyll was completely removed from the tissue at room temperature.

Plant tissue was fixed in 50% (*v*/*v*) ethanol, 5% (*v*/*v*) acetic acid, and 3.7% (*w*/*v*) formaldehyde at room temperature for 16 h. Tissue was dehydrated in a graded ethanol and xylene series and embedded in Paraplast Plus (Oxford Labware, St. Louis, MO, USA). Sections were sliced longitudinally or horizontally to 5–10 μm thickness using a microtome, and attached to Superfrost Plus microscope slides (Fisher Scientific, Pittsburgh, PA, USA).

### 4.7. Immunoblot Analysis of SLENDER RICE 1 (SLR1)

An antibody against SLR1 and an anti-ACTIN polyclonal antibody were purchased from Cosmo Bio (Cat. No. CT-NU-001-1; Tokyo, Japan) and Agrisera (Cat. No. As13 2640; Vännäs, Sweden), respectively. *OsPP2C08*-OX and wild-type (WT) seedlings were grown on half-strength MS medium containing GA_3_ or ABA. Whole shoots were ground in liquid N_2_, and total protein was extracted using an extraction buffer containing 0.05 M Tris–HCl (pH 7.4), 0.2% (*w*/*v*) SDS, 5% (*v*/*v*) glycerol, 1.5% (*v*/*v*) Triton X-100, 1% (*v*/*v*) β-mercaptoethanol, 1 mM EDTA, 1 mM dithiothreitol, and 1X complete Mini EDTA-free Protease Inhibitor (Roche, Rotkreuz, Switzerland). Immunoblotting was performed as described previously [24]. Peroxidase activity was detected using SuperSignal West Femto Maximum Sensitivity Substrate (Pierce, Rockford, IL, USA). Proteins were detected with Fusion SL2 (Vilber Lourmat, Eberhardzell, Germany).

### 4.8. Transcriptome Analysis

For transcriptome analysis, total RNA was prepared from 14-day-old seedlings grown on half-strength MS medium. Total RNA was extracted from shoots of OsPP2C08-overexpressing seedlings and control Dongjin seedlings with an RNeasy Plant Mini Kit (Qiagen, Hilden, Germany). Quality control was conducted with an Agilent Technologies 2100 Bioanalyzer (Agilent Technologies, Santa Clara, CA, USA). Sequencing libraries were prepared using a TruSeqRNA Sample Prep Kit v2 (Illumina, San Diego, CA, USA), following the manufacturer’s instructions. Library sequencing was performed using a HiSeq 4000 system (Illumina, San Diego, CA, USA), generating single-end 101-bp reads. Trimmed reads were mapped to IRGSP (v. 1.0) and assembled into transcripts. The read counts were determined using the StringTie program, then normalized with the DESeq2 program. All aligned reads to transcripts were normalized by transforming to fragment per kilobase of transcript per million mapped reads (FPKM), then to log2 fold-change before comparisons.

## Figures and Tables

**Table 1:** Expression profiles of GA metabolic pathway genes in WT and OsPP2C08-OX plants.

		Gene ID	WT-Mean *	WT-STDEV *	OX-Mean *	OX-STDEV *	Fold Change of OX/WT
*GA20ox*	*OsGA20ox1*	Os03g0856700	6.387454	0.03598653	5.953094	0.041642482	−1.35125
	*OsGA20ox2 (SD1)*	Os01g0883800	4.000492	0.14224135	3.644319	0.131228565	−1.28352
	*OsGA20ox4*	Os05g0421900	5.403216	0.28699917	7.934473	0.125858956	5.752747
*GA3ox*	*OsGA3ox2 (D18)*	Os01g0177400	6.607055	0.07167825	6.690712	0.095721887	1.058955
*GA2ox* (I)	*OsGA2ox3*	Os01g0757200	8.866322	0.25283603	9.214727	0.112922287	1.273661
	*OsGA2ox4*	Os05g0514600	6.277192	0.1829343	4.578638	0.125073243	−3.25041
*GA2ox* (II)	*OsGA2ox1*	Os05g0158600	6.667921	0.06508819	6.4263	0.130932063	−1.18283
*GA2ox* (III)	*OsGA2ox6*	Os04g0522500	7.123396	0.28365293	6.383509	0.079399404	−1.66837
*CPS*	*OsCPS*	Os02g0278700	7.044363	0.06118494	6.434432	0.158792429	−1.52498
*KS*	*OsKS*	Os04g0611800	9.16339	0.09306083	8.827319	0.090157625	−1.26251
*KO*	*OsKO/CYP701A (D35)*	Os06g0570100	9.297471	0.13797206	9.716885	0.088412978	1.337714
*KAO*	*OsKAO/CYP88A5*	Os06g0110000	6.218438	0.19921268	7.706477	0.121483696	2.80157
*GA16, 17ox*	*CYP714D1 (EUI)*	Os05g0482400	6.432289	0.30009856	6.971563	0.031743112	1.450763

* The mean values for three biological replicates were calculated.

## Data Availability

The original RNA seq data is available at NCBI’s Sequence Read Archive (SUB12982284).

## Supplementary Materials

The following supporting information can be downloaded at: https://www.mdpi.com/article/10.3390/ijms241310821/s1.
