# Supplementary material for: PROTEIN PHOSPHATASE 2C08, a Negative Regulator of Abscisic Acid Signaling, Promotes Internode Elongation in Rice
Source: Int J Mol Sci. 2023 Jun 28;24(13):10821. doi: 10.3390/ijms241310821 (PMC10341765; doi:10.3390/ijms241310821)
Supplement: Supplementary file 1 [file ijms-24-10821-s001.zip › ijms-2456062-supplementary.pdf]

Supplementary Materials:

Table S1. Primer sequences for qPCR analysis

Transcript	Direction	Sequence (5′ to 3′)
OsPP2C08	Forward	GGCTGTGGGATGTGGTATCC
	Reverse	CGTTCTACTCCTCAGCCGTC
OsRAB16	Forward	CATGGACAAGATCAAGGAGAAGC
	Reverse	CTTATTATTCAGGAAGGTGACGTGG
OsLEA03	Forward	GCCGTGAATGATTTCCCTTTG
	Reverse	CACACCCGTCAGAAATGCTCC
OsKAO	Forward	CTTCGCAAGAGCAAAGGCTG
	Reverse	TCCACCCCTTGGGGATAAGA
OsGA20ox4	Forward	AGTTCTTCGAGGACAGCAGC
	Reverse	CGATGTTGACGACGAACGC
OsGA2ox4	Forward	GCAGCTCATCTCCATCCTCC
	Reverse	CTCGACTTGAGCTTGTTGGC
OsUbi05	Forward	CGCCGTGCTCCAGTTCTACAAGG
	Reverse	TCCTTCCTTACTTCCGCCCCCA

Table S2. Days to 50% flowering of WT and OsPP2C08-OX plants

	Seed disinfection	Transplanting in paddy field	flowering
WT	April 30	June 6	August 21
OsPP2C08-OX	May 2nd	June 6	August 19

A

B

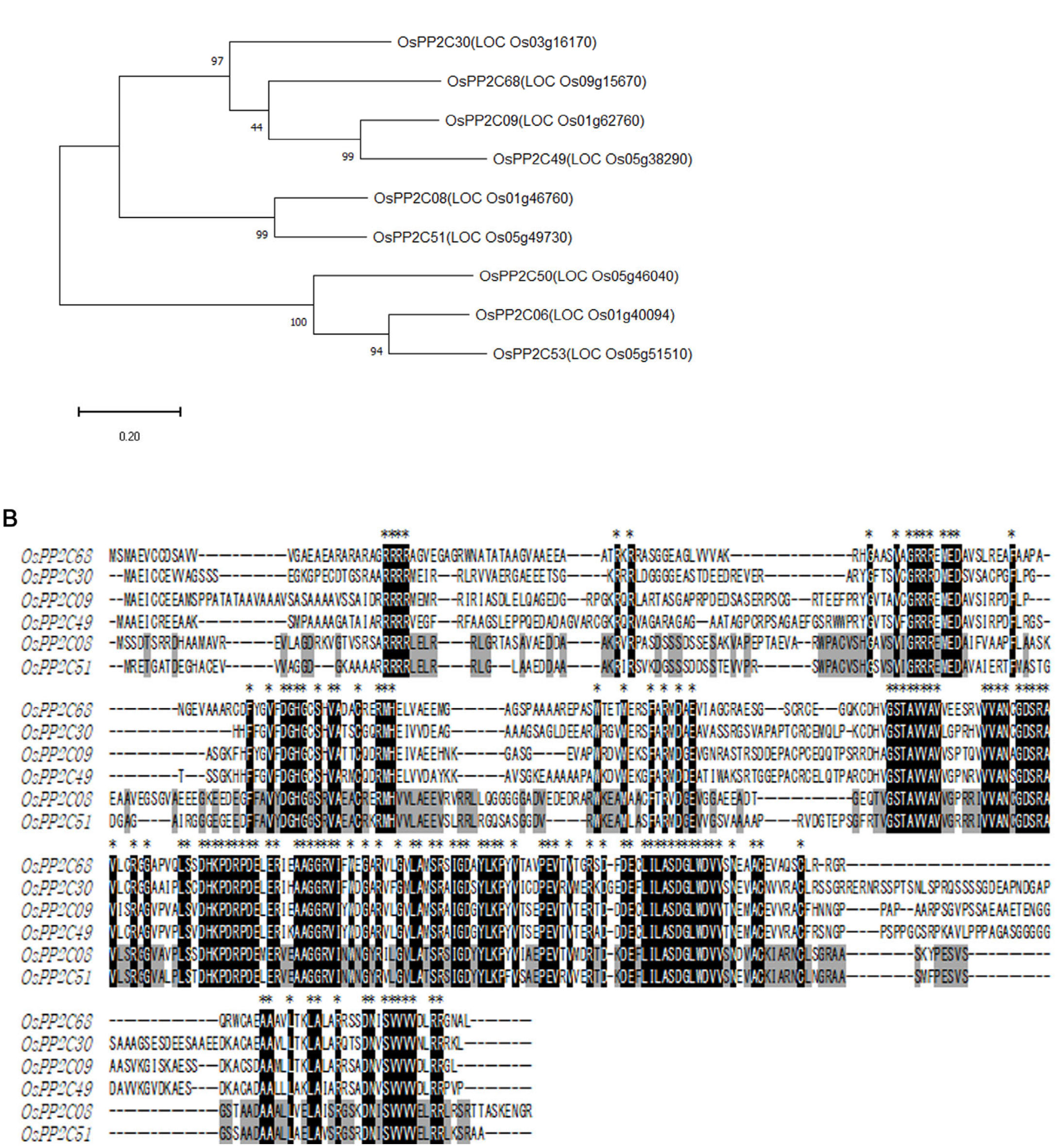

Figure S1. Amino acid sequence comparison of Clade A protein phosphatase 2Cs (PP2Cs). A. Maximum likelihood phylogenetic tree with 1,000 bootstrap replications based on the Clade A PP2Cs in rice. The deduced amino acid sequences were aligned using ClustalW, and the phylogenetic tree was drawn using the maximum likelihood method. Bootstrap values are shown at the nodes as a percentage of 1,000 replications. The tree is drawn to scale, with branch lengths, measured in the number of substitution per site. B. Alignment of amino acid sequences of rice Clade A PP2Cs. Black boxes indicate conserved residues in rice PP2CAs. Gray boxes indicate conserved residues only between OsPP2C08 and OsPP2C51.
